# Characterization and Analysis of Collective Cellular Behaviors in 3D Dextran Hydrogels with Homogenous and Clustered RGD Compositions

**DOI:** 10.3390/ma12203391

**Published:** 2019-10-17

**Authors:** Zheng Wang, Xiaolu Zhu, Ruiyuan Zhang

**Affiliations:** 1 College of Mechanical & Electrical Engineering, Hohai University, Changzhou 213022, China; wangzhengd0@163.com (Z.W.); ZRYBrian@163.com (R.Z.); 2 Jiangsu Key Laboratory of Special Robot Technology, Hohai University, Changzhou 213022, China; 3 Changzhou Key Laboratory of Digital Manufacture Technology, Hohai University, Changzhou 213022, China

**Keywords:** glycan, maleimide-dextran hydrogel, cell–substrate adhesion, 3D culture

## Abstract

The interactions between substrate materials and cells usually play an important role in the hydrogel-based 3D cell cultures. However, the hydrogels that are usually used could not be parametrically regulated, especially for quantitatively regulating the spatial distribution of the adhesion sites for cells in 3D. Here, we employed the semisynthetic hydrogel consisting of maleimide-dextran, Arg-Gly-Asp (RGD) peptides, and cell degradable crosslinkers to biochemically characterize the evolutionary behaviors of NIH–3T3 fibroblasts and C2C12 cells in 3D. Moreover, by comparing the cell-adhesive efficacy of 3D dextran hydrogels with four different RGD clustering rates, we explored the underlying regulation law of C2C12 connections and 3T3 aggregations. The results showed that mal-dextran hydrogel could promise cells stable viability and continuous proliferation, and induce more self-organized multicellular structures relative to 2D culture. More importantly, we found that RGD-clustered mal-dextran hydrogel has the advantage of enhancing C2C12 cell elongation and the breadthwise-aggregated connection, and promoting the 3T3 cell aggregating degree compared to that with homogenous RGD. Further, the advantages of RGD clustering hydrogel could be amplified by appropriately reducing RGD concentration. Such RGD-composition controllable mal-dextran hydrogel can function as a regulator of the collective cellular behaviors, which provides useful information for quantitatively designing the tailored hydrogel system and exploiting advanced biomaterials.

## 1. Introduction

In vitro cell culture has always been important in the biomedical field for decades [1,2]. With the development of various biomaterials, it is more convenient for us to construct tailored in vitro cell culture models by tuning the composition or configuration of the biomaterials for studying the physiological characteristics of cells deeply and understanding the inner mechanisms clearly [3,4]. According to the dimensions of the interface between cell and substrate materials, the mainstream in vitro cell culture techniques are mainly divided into 2D culture and 3D culture [5,6,7]. In 2D culture, the surface-modified substrate usually provides an opening space for cells to attach on, and cells are immersed with the culture medium directly [8]. Different substrate materials could induce distinguishing growth performances of cells [6,8]. 2D cell culture is a necessary method for in vitro cell culture, yet it still has some limitations for mimicking an in vivo microenvironment [9]. A 3D cell culture using hydrogel as the 3D substrate material could construct the suitable extracellular matrix (ECM) for cells [10,11], which could more realistically mimic native biochemical and biomechanical microenvironments compared with 2D culture [9,12,13]. The configuration of substrate materials for cells usually plays an important role in the research on 2D and 3D cultures, because the specifically designed functionalized biomaterials could influence the cellular behaviors via the microenvironments in multiple aspects [14,15]. To provide a healthy and adjustable environment for cells in 3D culture requires hydrogels with high hydrophilicity, porosity, variable mechanical properties, and good biocompatibility [16,17,18]. To meet these requirements for the 3D hydrogel scaffolds, numerous natural and synthetic hydrogels have been fabricated and tested, including materials such as dextran, hyaluronic acid, alginate, poly (ethylene glycol), and poly (vinyl alcohol) [19,20,21,22]. Mostly, their physical and chemical properties can be regulated to satisfy the requirements of cell growth, such as cellular adhesion, migration, proliferation, and differentiation [20,23,24]. Some hydrogel materials showed potential in regulating cell growth parametrically and quantitatively [25,26]. Dextran hydrogel, as one of the semisynthetic hydrogels, can satisfy the requirements above [27,28,29,30,31]. Dextran is a linear polysaccharide consists of α-1,6 linked D-glucopyranose, and it can be modified with diverse functional groups for promoting further cell behaviors [32,33]. All these can help fabricate variants of dextran hydrogel with ideal mechanical and biochemical properties and construct more realistic 3D culture models by better mimicking the in vivo microenvironment [33,34,35,36].

Further, improved methods of fabricating such hydrogel matrices can help direct single-cell and collective cellular behaviors, by which researchers can pierce into the cell–ECM interactions more conveniently [19,37,38,39]. For example, RGD peptides were known as the anchors of cellular adhesion, and they function as the connection between scaffold polymers and integrin spanning the cell membrane. Integrin clustering plays an important role in the activation of the signal transduction pathway that mediates cellular activities such as reorganization of the intracellular cytoskeleton, regulation of the growth factors, and the control of ion channels [40]. The spatial distribution of RGD peptides in the scaffolds can usually influence cell adhesion dynamics and motility. Researches in 2D matrices have shown that RGD spatial distribution may affect cell adhesion on the nanoscale [41,42]. However, the specific impact laws of the specific RGD distribution on the collective cell behaviors in 3D hydrogels, especially for dextran hydrogel, have not been intensively and fully characterized and analyzed, which hinders the in-depth exploration for the underlying mechanism.

In this study, we fabricated the maleimide-dextran hydrogel with homogenous and clustering distributions of RGD peptides, and employed the 3D dextran hydrogels parametrically designed with homogenous and clustered RGD compositions to explore the flexible and quantitative regulation of the substrate materials influencing the collective cellular behaviors. We studied the growth characteristics of NIH–3T3 fibroblasts (NIH denotes National Institutes of Health) and C2C12 cells within 3D dextran hydrogel with homogenous composition, and discussed the effect of the hydrogel materials on cellular behaviors relative to that on 2D substrates. Further, we fabricated the dextran hydrogels with four different clustering degrees of RGD compositions and measured their cell-adhesion efficacy by comparing cellular performance. The property of the RGD-homogenous hydrogel was characterized through SEM imaging and rheological analysis. Its biocompatibility was quantitatively characterized by cytotoxicity assay, survival rate analysis, and proliferation measurement. The RGD-clustered hydrogel was quantitatively assessed via measuring the hydrogel’s cell-adhesion efficacy, observing the evolutionary cellular morphology and the distribution of F-actins inside cells in 3D. The results showed that RGD-clustered hydrogel has the advantage of enhancing cell elongation and the connection or aggregating degree of cells, compared to the sample with homogenous RGD. Varying the degree of RGD-clustering in hydrogels could serve as a stable approach for modulating the cellular growth behaviors. This study on the impact of 3D dextran hydrogels with homogenous and clustered RGD compositions on cellular behaviors provides useful information for quantitatively designing the tailored hydrogel system and lays a foundation for quantitatively investigating the cell–biomaterial interactions in tissue morphogenesis.

## 2. Materials and Methods

### 2.1. 3D Dextran Hydrogel

**Figure 1:**
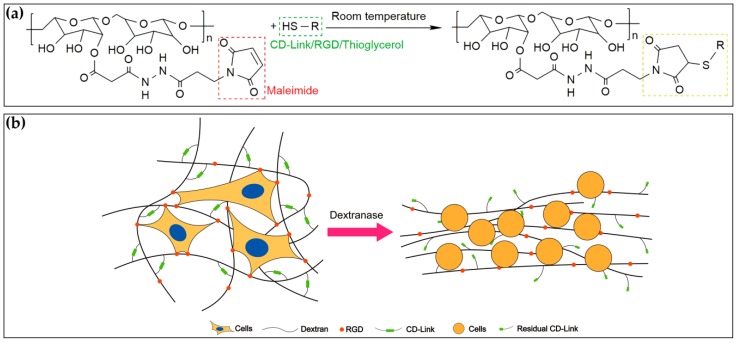
Fabrication and degradation of 3D homogenous mal-dextran hydrogels. (**a**) Maleimide–thiol reaction formula: maleimide groups were on dextran and thiol groups (-SH) were on CD-Link (thiol-containing crosslinker), RGD peptides, and thioglycerol; (**b**) Degradation of the formed dextran hydrogel.

The dextran hydrogel (Cat No: FG91-1, Cellendes, Reutlingen, Germany) was prepared with deionized (DI) water, 10-fold concentrated buffers (10 × CB, pH 5.5), maleimide-dextran (thiol-reactive polymer), and cell degradable crosslinkers (CD-Link, thiol-containing crosslinker). It is a quick-forming hydrogel that can be fabricated in approximately 3–5 min. The time of gel formation is mainly dependent on the concentration of thioether and the pH value of the buffer system [43]. The mechanical properties of hydrogel, such as stiffness and viscosity, correspond to the proportion of gel components. In this study, the crosslinking process happened between thiol-reactive maleimide-dextran and thiol-containing CD-Link (Figure 1a). Gel stiffness was positively correlated to the concentration of reacted maleimide groups, and this concentration was defined as the crosslinking strength in this study. Additionally, the thiol-containing RGD peptides (Cat No: 09-P-001, Cellendes, Reutlingen, Germany) were used to functionalize the maleimide-dextran for cell adhesion, and thioglycerol (Cat No: T10-3, Cellendes, Reutlingen, Germany) was used to maintain the equal final concentration between maleimide and thiol groups (Figure 1a). Such 3D dextran hydrogel can be easily degraded by the dextranase (Figure 1b). The sequence of RGD peptide is Acetyl-Cys-Doa*-Doa-Gly-Arg-Gly-Asp-Ser-Pro-NH_2_ (*: Doa: 8-amino-3,6-dioxaoctanoic acid).

### 2.2. Cell Preparation

NIH–3T3 fibroblasts and C2C12 cells were cultured on the substrate surface of the 100 mm × 20 mm cell culture dish (Cat No: 704001, Nest Biotechnology Co., Ltd., Wuxi, China). The components of the culture medium were 1% penicillin–streptomycin solution (Cat No: SV30010, HyClone, GE Healthcare Life Sciences, South Logan, UT, USA), 10% fetal bovine serum (Cat No: 13011-8611, EVERY GREEN, Zhejiang Tianhang Biotechnology Co., Ltd., Hangzhou, China) and 89% DME/F-12 (Cat No: SH30023.01, HyClone, GE Healthcare Life Sciences, South Logan, UT, USA). The inside environment of the cultivator was 37 °C and 5% CO_2_. Cells were plated at 1 × 10^6^ in each dish, and were passaged every 3 days. In this study, the passage number of NIH-3T3 fibroblasts was P7 – P10, and that of C2C12 was P14 – P17. To harvest cultured cells from the substrate, replace the culture medium with 2 mL of phosphate-buffered saline (PBS) (Cat No: SH30256.01, HyClone, GE Healthcare Europe GmbH, Freiburg, Germany) and 1 mL of trypsin 0.25% (1X) solution (Cat No: SH30042.01, HyClone, GE Healthcare Life Sciences, South Logan, UT, USA). Cells were incubated in the cultivator for 4 min before being detached from the substrate. Then, the detached cells were transferred into a 15-mL centrifuge tube, centrifuged (3T3, 1000 r/min, 3 min and C2C12, 1000 r/min, 6 min), resuspended in fresh culture medium, and counted with a Metallized Hemacytometer (Cat No: 1483, HAUSSER SCIENTIFIC, Horsham, PA, USA).

### 2.3. RGD-Homogenous Hydrogel Fabrication

To obtain the precursor solution, DI water, 10 × CB (pH 5.5), maleimide-dextran, RGD peptides, and thioglycerol were added into a reaction tube in proportion and mixed thoroughly. Then, the precursor solution was incubated for 5–10 min in room temperature for a complete reaction (Figure 1a). CD-Link was placed onto the bottom of the wells of a 96-well plate. The cell suspension was added into the precursor solution and mixed evenly. The final cell density in the gel was set at 5 k/µL. Then, the cell-containing precursor solution was transferred into the wells containing CD-Link and mixed two times quickly and pliably. During this operation, it was critical to avoid air bubbles, which would influence the later observation and imaging. The hydrogel was completely formed in 3–5 min at room temperature. The sample was covered with fresh culture medium and incubated in the cultivator. The medium was renewed after cultivation of 2 h. Medium was changed every two days during cultivation. The volume of each hydrogel sample for cell culture was always maintained at 30 μL, and the reagents were kept on ice. As the control, 3T3 and C2C12 cells were also culture on tissue culture polystyrenes (TCPS) respectively.

### 2.4. RGD-Clustered Hydrogel Fabrication

**Figure 2:**
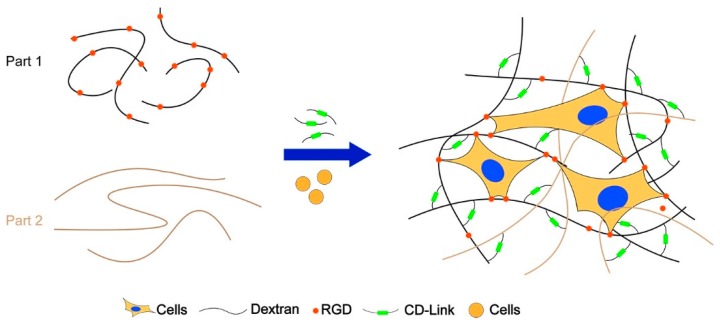
Illustration of fabricating 3D dextran hydrogels with different clustered RGD compositions.

The fabrication method of homogenous RGD-functionalized dextran hydrogel has been given in Section 2.3. This section gave the improved method for regulating the clustering rate (or homogenous level) of RGD distribution in dextran hydrogel. When preparing the precursor solution, maleimide-dextran was divided into two parts for the mixture (Figure 2). DI water, 10 × CB (pH 5.5), the first part of maleimide-dextran, and RGD peptides were added into a reaction tube in proportion, mixed thoroughly, and incubated for 5–10 min in room temperature. After the reaction was completed, the second part of maleimide-dextran and thioglycerol were added into the reaction tube, mixed thoroughly, and incubated for 5–10 min in room temperature. Four different cases were tested, and the related parameter values were listed in Table 1. The final cell density in the gel was set at 2.5 k/µL. The next steps for hydrogel fabrication followed the contents in Section 2.3.

### 2.5. SEM Imaging

The hydrogel samples were imaged with a scanning electron microscope (SEM) (Sigma-500, Zeiss, Oberkochen, Germany). The samples were cut out to indicate their internal surfaces. The working voltage was set at 10.0 kV, and the working distance was 9.00 mm. Gel images magnified 5000 and 20,000 times have been obtained.

### 2.6. Rheology Measurement

The elastic modulus (G’) and viscous modulus (G’’) of hydrogel with three different crosslinking strengths were measured with a plate-to-plate rheometer (Kinexus Pro, Malvern, UK). The volume of each hydrogel sample was 90 μL, and the gap distance was set at 0.2 mm. The complex shear strain was 1%, and the frequency ranged from 0.1 to 10 Hz with the temperature inside the humid hood set at 37 °C.

### 2.7. Live/Dead Test

A LIVE/DEAD Viability/Cytotoxicity Kit (Cat No: L3224, Thermo Fisher Scientific, Eugene, OR, USA) was used for testing cellular viability. To get the live/dead dye solution, 3.6 μM ethidium homodimer-1 and 2.8 μM calcein acetoxymethyl ester (calcein-AM) solution were obtained by dissolving them in 1 mL of Dulbecco’s modified Eagle’s medium/Ham’s nutrient mixture F-12 (DME/F-12). The medium was moved out from the well, and the sample was washed with PBS two times. Solution (150 μL) was added into the well, and the sample was incubated in dark at 37 °C for 25 min. Then, the sample was washed with PBS three times, 5–10 min each time, and observed and imaged under the laser with PBS left in the well. Cells were observed by an inverted microscope (Olympus IX73, Olympus Corporation, Tokyo, Japan), and the images were taken with a digital sCMOS camera (HAMAMATSU C11440-42U30, Hamamatsu Photonics K.K., Hamamatsu, Japan). With the help of ImageJ (National Institutes of Health, Bethesda, MD, USA), we counted the number of living and dead cells in the images. In 3D culture, the sample numbers of survival rates of 3T3 on day 0, day 3, day 6, and day 9 were 9, 7, 7, and 11; the sample numbers of survival rates of C2C12 on day 0, day 3, day 6, and day 9 were 4, 7, 4, and 10, respectively. In 2D culture, the sample numbers of survival rates of 3T3 on day 0, day 3, day 6, and day 9 were 5, 5, 5, and 5; the sample numbers of survival rates of C2C12 on day 0, day 3, day 6, and day 9 were 5, 5, 5, and 5. We conducted the calculation for the mean and standard deviation of the data.

### 2.8. Bright Field Imaging

With an inverted microscope and the digital sCMOS camera, we took the images of 3T3 and C2C12 under the bright field. Specially, the filopodia and lamellipodia of cells in 2D and 3D were imaged, and we counted the amount of filopodia of 3T3 in 2D, 3T3 in 3D, C2C12 in 2D, and C2C12 in 3D respectively, and the cell sample numbers were 57, 35, 32, and 53, correspondingly. Additionally, in this study, cellular behaviors such as spreading, sprouting, and both were recognized as the symbol of cell adhering.

### 2.9. F-Actin Staining

Alexa Fluor™ 546 phalloidin (Cat No: A22283, Invitrogen, Thermo Fisher Scientific, Eugene, OR, USA) was used for F-actin staining. The medium was moved out from the well, and the sample was washed with PBS three times. Cells were fixed with 4% formaldehyde (Cat No: 28908, Thermo Scientific, Rockford, IL, USA) solution in PBS in the dark at room temperature for 45 min. Then, the sample was washed with PBS three times. Then, cells were extracted with a solution of 0.1% Triton X-100 (Cat No: X100-5ML, SIGMA-ALDRICH, St. Louis, MO, USA) in PBS for 5 min. Then, the sample was washed two or more times with PBS. One kit of Alexa Fluor™ 546 phalloidin was dissolved in 1.5 mL of methanol (Cat No: M116128-1L, Aladdin Industrial Corporation, Shanghai, China) to get the 40× stock solution. Then, 10 μL of stock solution was diluted in 200 μL of PBS for each well to be stained. To reduce the nonspecific background, 1% bovine serum albumin (BSA) (Cat No: 37525, Thermo Scientific, Rockford, IL, USA) was added into the staining solution. The staining process lasted 20 min in dark at room temperature. Then, the sample was washed with PBS at least three times.

### 2.10. DAPI Staining

The fixed and permeabilized sample was washed with PBS first. To get the dye solution, 4′, 6-diamidino-2′-phenylindole, dihydrochloride (DAPI) (Cat No: 62247, Thermo Scientific, Dreieich, Germany) stock solution was diluted to 138 ng/mL in PBS. Then, 300 µL of DAPI staining solution was added into each well, and the sample was incubated in dark at room temperature for 5 min. Then, the sample was washed with PBS at least three times.

### 2.11. LSCM Imaging

The F-actin and DAPI stained samples were imaged by a laser scanning confocal microscope (LSCM) (LSM-710, Zeiss, Oberkochen, Germany). Samples were transferred onto the microscope cover glass (Cat No: 12-541-B, Fisher Scientific, Waltham, MA, USA) and soaked with PBS. Z-axis accuracy was set at 0.56 μm with an image model of 16 bit and pixel dwell time of 0.79 μs.

### 2.12. Nucleus Circularity Measuring Method

The ellipses with the circularity ranging from 0.1 to 1 at an interval of 0.02 were drawn by MATLAB (Matlab R2019, MathWorks Inc., Natick, MA, USA, trial version). These ellipses were then compared with the nucleus in the image of DAPI staining results. Once finding the ellipse with the closest shape to the measured nucleus, the circularity value of that ellipse was taken as the measured nucleus’s circularity value. By using the same method, the circularity of the nucleus in images was estimated, and the mean and standard deviation of data was then calculated and charted. The cell sample numbers used for assessing nucleus circularity of 3T3 in 2D, 3T3 in 3D, C2C12 in 2D, and C2C12 in 3D were 45, 45, 45, and 45. Circularity was defined as follow:

(1) $$Circularity=\frac{4\pi S}{L^{2}}$$

S was the area, and L was the perimeter of the single nucleus.

### 2.13. Gel Degradation

The medium was moved out from the microwell. The sample was covered with 300 μL of a 1:20 dilution of dextranase (Cat No: D10-1, Cellendes, Reutlingen, Germany) in culture medium and incubated at 37 °C for 30 min. Gels could be dissolved faster if they were cut into pieces. After the degradation of the gel, the cell suspension was centrifuged, and cells were resuspended in fresh culture medium. We counted the number of cells with a Metallized Hemacytometer. The gel sample numbers for counting 3T3 cells on day 3, day 6, and day 9 were 3, 3, and 3; the gel sample numbers for counting C2C12 cells on day 3, day 6, and day 9 were 3, 3, and 3.

### 2.14. Data Statistics

The data were presented by mean ± standard deviation (Mean ± SD). Two-sample Student’s t-Test was used to analyze the significant difference of the data in Origin software (OriginPro 2018 v9.5 64-bit, OriginLab Corporation, Northampton, MA, USA, trial version). The upper limit value of significance level was set as *p* < 0.05. All the experiments were repeated at least three times.

## 3. Results

### 3.1. Microgeometry and Rheological Properties of Dextran Hydrogel

**Figure 3:**
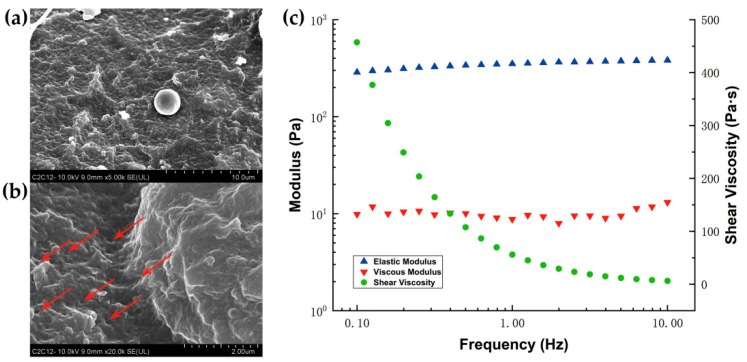
SEM images and viscoelasticity of the 3D homogenous dextran hydrogels. The main parameters of dextran hydrogel were crosslinking strength = 2 mM and RGD = 300 μM; (**a**) Image of 3D dextran hydrogel with 5000 times magnification under SEM; (**b**) Image of 3D dextran hydrogel with 20,000 times magnification under SEM; (**c**) The elastic modulus (G′), viscous modulus (G″), and shear viscosity (complex component) of dextran hydrogel.

We imaged the microgeometry of the 3D dextran hydrogel by scanning electron microscopy (SEM). The sample was cut out to image its internal surfaces. The results showed that the internal surface of the gel was pleated (Figure 3a). It indicated that 3D dextran hydrogel can provide a rough contact surface for cells in it. Some multipore structures were marked with the arrows in Figure 3b. The elastic modulus (G′), viscous modulus (G″), and shear viscosity (complex component) of the dextran hydrogel with different crosslinking strengths were measured with a plate-to-plate rheometer at 37 °C. Results showed that the G′ and G″ separately settled on the different orders of magnitude over the entire ranging of measured frequencies (0.1–10 Hz) (Figure 3c), and the value of G′ and G″ is correlated to the mechanical properties of hydrogel. The average value of G’/G’’ was lower than 0.1, which indicated that the elastic property of dextran hydrogel was more pronounced than its viscosity. Dextran hydrogel, used in this study, can be fabricated with different stiffness by allocating the proportion of maleimide-dextran, CD-Link, and RGD peptides. The crosslinking strength of dextran hydrogel was defined as the concentration of maleimide groups from dextran crosslinked by thiol groups from CD-Link.

### 3.2. Cytotoxicity and Proliferation Measurement for RGD Homogenous Dextran Hydrogel

**Figure 4:**
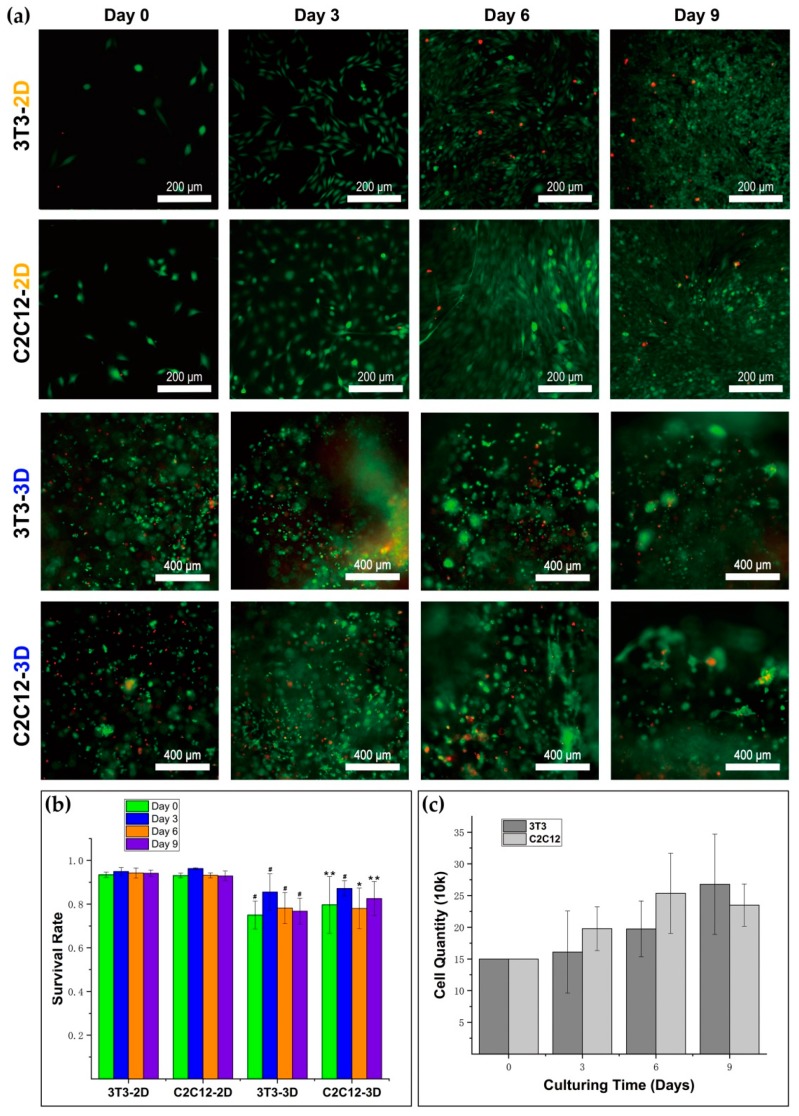
Viability and proliferation of cells in 2D and 3D. (**a**) Live/dead test conducted on 3T3 and C2C12 in a 2D petri dish (TCPS control) and 3D dextran hydrogel; (**b**) Survival rates of 3T3 and C2C12 from day 0 to day 9. The data were presented by mean ± SD; (**c**) Numbers of 3T3 and C2C12 cells in 3D from day 0 to day 9. The data were presented by mean ± SD; * *p* < 0.05, ** *p* < 0.03, and # *p* < 0.01 versus the corresponding 2D control samples.

A live/dead test has been conducted on 3T3 and C2C12 on day 0, day 3, day 6, and day 9, respectively with initial cell density of 5000/μL. The results are shown in Figure 4. Green spots represent living cells, and red spots represent dead cells. The results showed that NIH–3T3 fibroblast and C2C12 cells in the 2D petri dish showed higher survival rates that those in the 3D dextran hydrogel with homogenous distributions of RGD peptides (Figure 4b). The number of green spots was larger than the number of red spots in the images (Figure 4a). It indicated that both 3T3 and C2C12 can keep viability in 3D dextran hydrogel for days. With the extension of culturing time, the ‘green’ cells appeared to aggregate, and grew into specific structures in the hydrogel (Figure 4a). This evolution indicated that cells in such hydrogel material can keep capacities of proliferation and grow into multicellular structures. The mean and standard deviation of the data were conducted (Figure 4b), including the cell survival rates of 3T3 and C2C12 respectively on day 0, day 3, day 6, and day 9. Both 3T3 and C2C12 kept a survival rate of over 75%. The survival rates of 3T3 and C2C12 increased over the first three days and were kept stable during the entire test.

The proliferation of 3T3 and C2C12 were studied by manual counting on day 3, day 6, and day 9, respectively. The initial cell quantity of each sample was 150,000. The results showed that the proliferation of 3T3 and C2C12 in 3D dextran hydrogel increased, but was hardly doubled during the cultivation of nine days (Figure 4c). Although 3T3 and C2C12 in the 3D hydrogel in a 96-well plate exhibited lower proliferation rates than the cells on 2D surface in a six-well plate with the same initial cell quantity (Supplementary Figure S4); the results also informed us, from other aspects, that such hydrogel material can hold 150,000 cells in 3D matrices in a microwell with a bottom area of 32 mm^2^ initially, and keep cells proliferating continuously. During cultivation in hydrogel, the proliferation of 3T3 and C2C12 showed different regularity. The proliferation of 3T3 fibroblasts increased continuously for 9 days (Figure 4c), while the proliferation of C2C12 was increased continuously and higher than that of 3T3 over the first 6 days, but it decreased obviously on day 9 (Figure 4c).

### 3.3. Cellular Morphology and Behaviors in RGD-Homogenous Dextran Hydrogel

**Figure 5:**
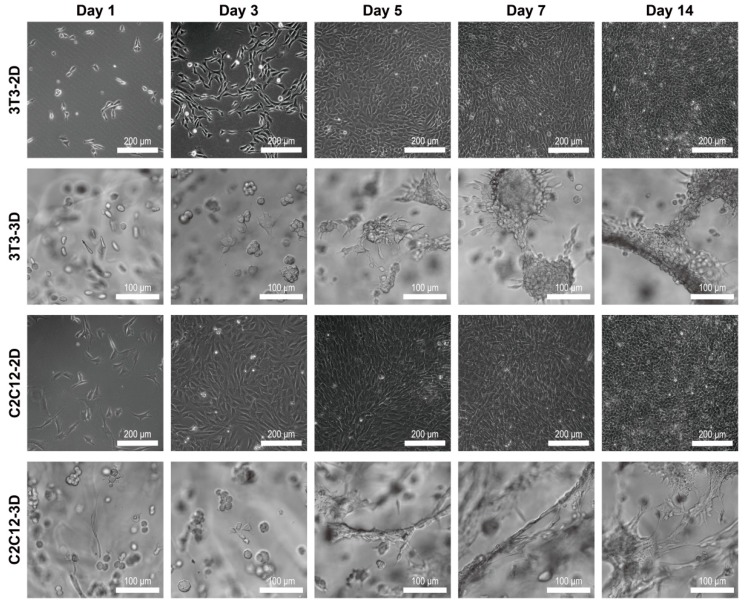
Bright-field images of 3T3 and C2C12 cells in a 2D petri dish and 3D RGD-homogenous dextran hydrogel from day 1 to day 14. The main parameters of dextran hydrogel were crosslinking strength = 2 mM and RGD = 300 μM.

**Figure 6:**
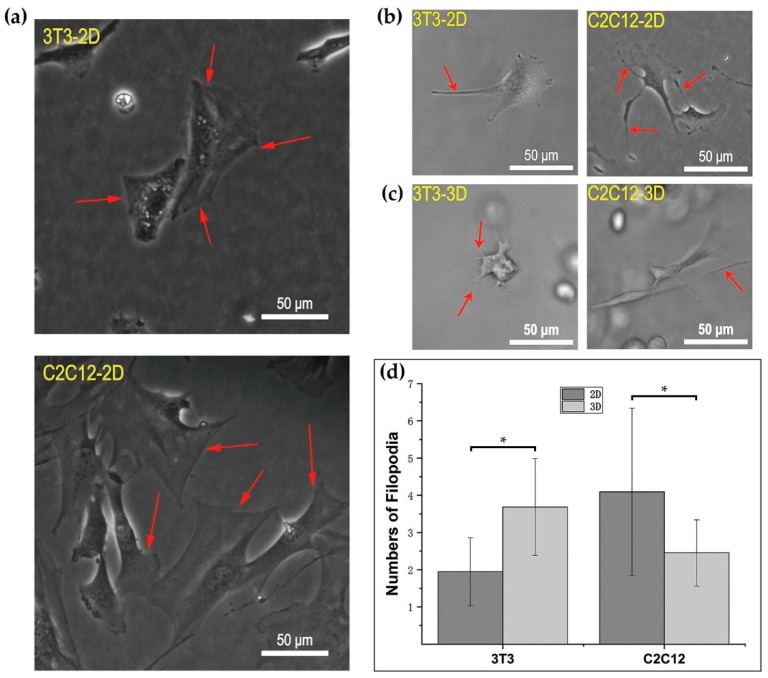
Lamellipodia and filopodia of cells in 2D dish and 3D dextran hydrogels. (**a**) Lamellipodia of 3T3 and C2C12 in 2D; (**b**) Filopodia of 3T3 and C2C12 in 2D; (**c**) Filopodia of 3T3 and C2C12 in 3D; (**d**) Numbers of filopodia of cells in 2D petri dishes and 3D dextran hydrogel. The data were presented by mean ± SD; * *p* < 0.05 versus the corresponding 2D control samples.

3T3 and C2C12 were cultured under the same treatments, including a cultivating environment of 37 °C, 5% CO_2_, and refreshing the culture medium every 2 days for 2 weeks. Images of 3T3 and C2C12 were taken on day 1, day 3, day 5, day 7, and day 14 regularly. Due to the different dimensions of the substrate provided by substrate materials, cells showed a distinguishing basic configuration in 3D dextran hydrogel and a 2D petri dish. Over 2 weeks of cultivation, 3T3 fibroblasts and C2C12 cells usually exhibited the plump and stereo shape in 3D, while the shape of cells in 2D tended to be more flattened (Figure 5). The sprouting and spreading of cells are the representative cellular behaviors reflecting cell–matrix interactions. In our observation, filopodia and lamellipodia were the two main cellular growing structures during sprouting and spreading. Lamellipodia are cytoskeletal protein actin projections on the front edge of the cell. Filopodia are slender cytoplasmic projections extending from the front edge of lamellipodia in migration cells. In 3D, most 3T3 and C2C12 cells were usually observed growing out some slender filopodia, while their lamellipodia were relatively inconspicuous in the hydrogel (Figure 6c). In 2D, both 3T3 and C2C12 cells had attachment, with their filopodia and lamellipodia adherent on the surface of petri dishes (Figure 6a,b). The polygon-shape of cells in 2D was mainly associated to the amount and morphology of their cellular lamellipodia (Figure 6a). With the extension of culturing time, cells occupied more space in or on the substrate material by continuous proliferation, connectivity, and migration. In 3D dextran hydrogel, patterns of 3T3 and C2C12 were exhibited as distributed structures; that is, isolated multicellular clumps were cross-connected by a path of chains composed of cells (Figure 5, 3T3-3D and C2C12-3D). In the 2D petri dish, patterns of cells usually kept an even cellular distribution and more random directions of cellular polarization. During the entire cultivation, the locations of 3T3 and C2C12 were almost isotropic (Figure 5, 3T3-2D and C2C12-2D).

Despite the same culturing conditions, 3T3 and C2C12 cells in 3D dextran hydrogel also exhibited distinguishing growth phenotypes. For these cells, the polarized state could be observed approximately 12 to 24 h after implantation initially. However, C2C12 cells presented greater degree of autologous stretching than 3T3 (Figure 5, Day 5 to Day 14, 3D). In addition, their rates of sprouting were distinct. For 3T3, it usually took 2–3 days to sprout mostly. Under the same conditions, that occurred on C2C12 after about 1–2 days of culturing. By comparison, the amount of filopodia of cells was changed when transferred from petri dishes into dextran hydrogel. Once spread in 3D hydrogel, the amount of filopodia of 3T3 was larger than that in the 2D petri dish (Figure 6d). In contrast, after being transferred from 2D to 3D, the amount of observable filopodia of C2C12 got reduced (Figure 6d). The main morphological characteristics of self-organization were cell clumps and cellular connections with a bigger scale. 3T3 cells tended to proliferate to form clumps. With continuous proliferation, the volume and number of 3T3 cell groups gradually increased. Connections between the clumps of 3T3 cells usually rose after that. The process started with a single cell to a mass; then, the cells were attracted to and connected with each other by growing out cells similar to a bridge (Figure 5, 3T3-3D). Both multicellular clumps and connecting structures were composed of dense cells. In contrast, C2C12 preferred to stretch itself longer and connect with each other first, and long chains or mesh structures were usually observed from C2C12 in 3D dextran hydrogel (Figure 5, C2C12-3D). In the process of connection, proliferation also occurred. After culturing for more than one week, it was common to observe that C2C12 cells of clumps extended around and connected with each other by slender bundles of stretched cells.

**Figure 7:**
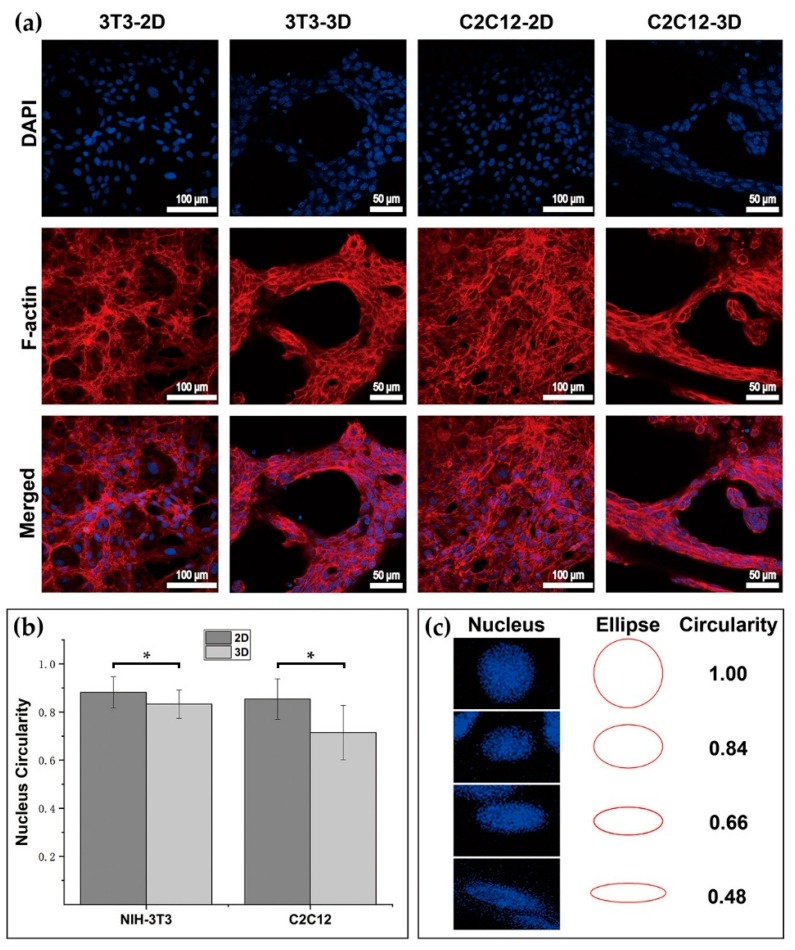
Cytoskeletal staining and analysis for cells in 2D and 3D. (**a**) DAPI and F-actin staining of 3T3 and C2C12 cells in 2D and 3D culture; (**b**) Circularity of cellular nucleus in 2D petri dishes and 3D dextran hydrogel. The data were presented by mean ± SD; * *p* < 0.05 versus the corresponding 2D control samples. (**c**) Demonstrations of estimating the circularity of cell nucleus.

F-actin and DAPI staining were conducted on cells in the 2D petri dish and 3D dextran hydrogel, respectively. Images of cellular nucleus and microfilament taken under the LSCM exhibited the multicellular structures of 3T3 and C2C12 in 2D and 3D. As different substrate materials, dextran hydrogel and the petri dish surface contributed distinguishing cytoskeleton and self-organization characteristics to the cells. Both 3T3 and C2C12 in 3D dextran hydrogel tended to grow into multicellular clumps with a densely packed nucleus, and the shape of their nucleus was influenced in the hydrogel materials. That is, the nucleus shape of 3T3 and C2C12 in hydrogel presented lower circularity than that of cells in dishes (Figure 7b,c). In addition, cells usually spread out on the bottom surface of the petri dish with the microfilaments incompact from each other. In contrast, 3T3 and C2C12 in dextran hydrogel presented more agglomeration and connectivity with the microfilaments squeezed tightly with each other (Figure 7a). Compared to the results in 2D, the growth of 3T3 and C2C12 in 3D dextran hydrogel was featured with more 3D interleaving structures. To be more specific, during the 14 days of cultivation, 3T3 grew out many banded structures with numerous cells gathered and self-organized together and distributing in the hydrogels. Some doughnut-shaped holes with various sizes were usually observed in the multicellular connection structures of 3T3 (Figure 7a, 3T3-3D). For C2C12 cells in 3D dextran hydrogel, the staggered ‘truss’ structures that can be found are typical of them. C2C12 cells in the same path tended to connect into a slender long chain, followed with proliferation. C2C12 cells in such chain structures tended to exhibit lower circularity of the cell nucleus (Figure 7a, C2C12-3D).

### 3.4. Cell-Adhesive Efficacy of RGD Clustering Dextran Hydrogels

**Figure 8:**
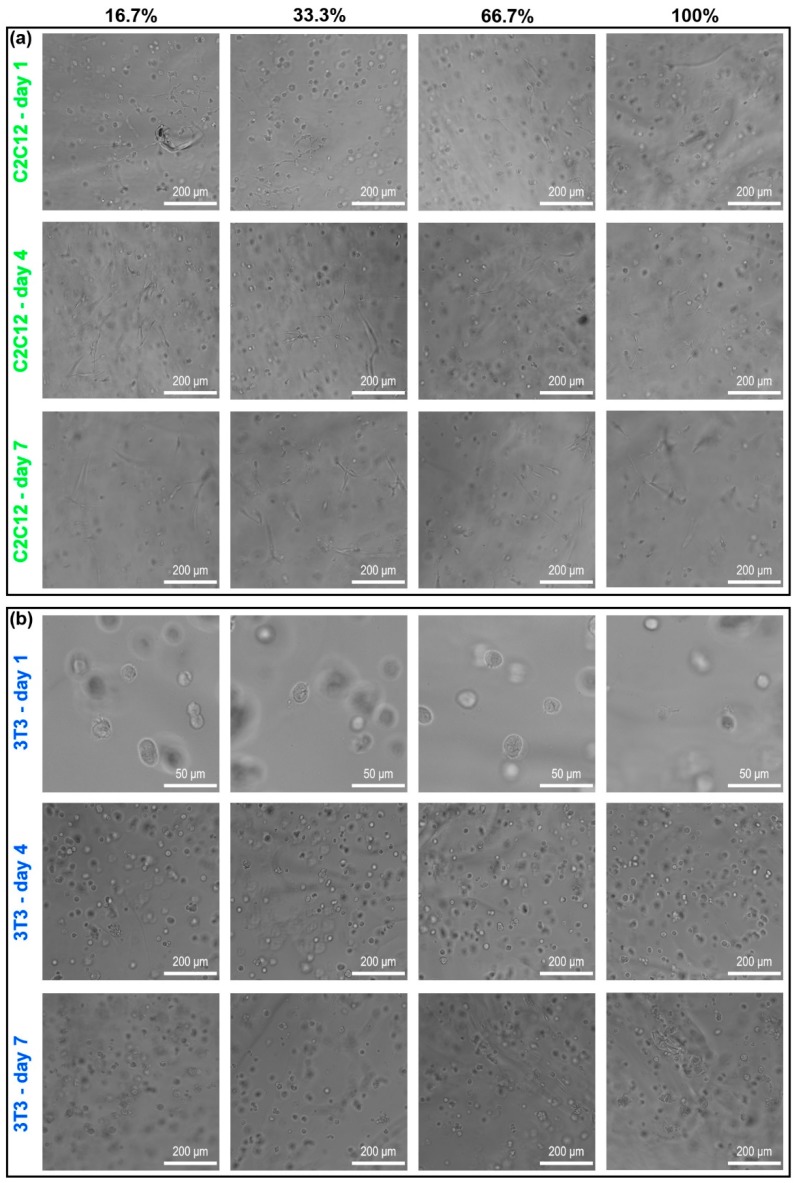
The images of C2C12 and 3T3 cells in four different RGD clustering cases under the bright field. The main parameters of dextran hydrogel were crosslinking strength = 2 mM and RGD = 50 μM. (**a**) C2C12 cells; (**b**) 3T3 cells. Cells were observed by an inverted microscope, and the images were taken with a digital sCMOS camera. The proportions of the mal-dextran reacted with RGD for four cases are 16.7%, 33.3%, 66.7%, and 100%, respectively.

Three-dimensional (3D) dextran hydrogels with four different RGD clustering compositions have been fabricated and tested for culturing NIH-3T3 fibroblasts and C2C12 cells for one week, respectively. The proportions of the mal-dextran that reacted with RGD (abbreviated to proportions of dextran with RGD) for four cases are 16.7%, 33.3%, 66.7%, and 100% respectively. According to the images under the bright field, C2C12 and 3T3 cells could normally spread and sprout in both RGD-homogenous and RGD-clustered dextran hydrogels (Figure 8a C2C12-day 1 and 8b 3T3-day 1). With the extension of culturing time, C2C12 cells gradually became more elongated and connected into the long branch structures (Figure 8a C2C12-day 4 and C2C12-day 7). Then, 3T3 cells tended to aggregate into clumps both in RGD-homogenous and RGD-clustered dextran hydrogels (Figure 8a 3T3-day 4 and 3T3-day 7).

**Figure 9:**
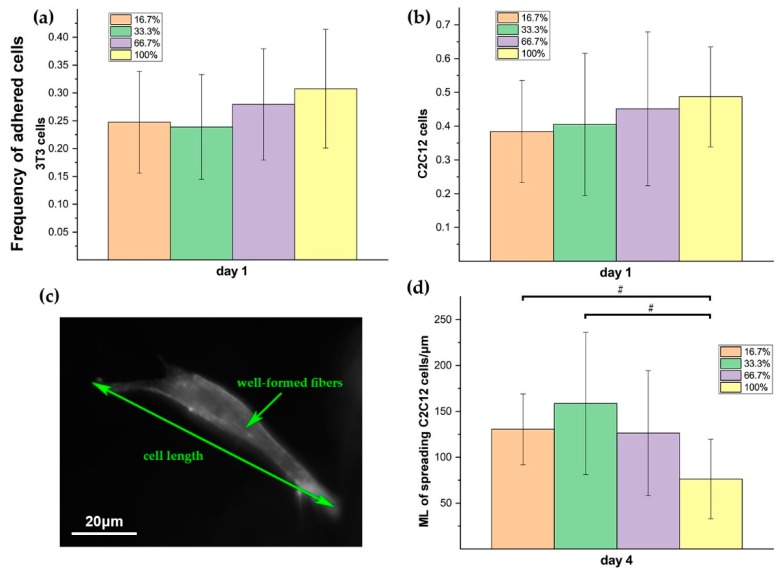
The impact of the composition configuration of 3D dextran hydrogels with clustered and homogenous RGD distribution on initial cell adhesion. The main parameters of dextran hydrogel were a crosslinking strength = 2 mM and RGD = 50 μM. (**a**) Adhesion rates of 3T3 cells on day 1 in groups 1–4; (**b**) Adhesion rates of C2C12 cells on day 1 in groups 1 – 4; (**c**) The well-formed fibers of adhered C2C12 cells were stained by phalloidin. (**d**) The maximum length (ML) of spreading C2C12 cells was measured on day 4 in groups 1–4. The measurement was conducted by ImageJ software. The data were presented by mean ± SD; # *p* < 0.01 versus the corresponding 3D homogenous samples.

We counted the adhered 3T3 and C2C12 respectively on day 1 to access the initial cell adhesive efficacy of 3D dextran hydrogels with different clustered RGD compositions. The results showed that for both 3T3 and C2C12, cells showed relatively higher average adhesion rates in 3D hydrogels with more homogenous RGD composition (Figure 9a,9b). Additionally, the adhesion rates of C2C12 cells were higher than those of 3T3 cells generally in most cases (Figure 9a,b). It can be observed under the bright field, cells could normally spread, sprout, and aggregate in these four cases. The specific performances varied on different cells, which have been partly mentioned in the homogenous case above. Further, the phalloidin staining results showed that cells with higher-level extension or elongation tended to appear as ‘well-formed’ F-actin skeletons, which means curves of intracellular microfilaments fit well with the postures of the cellular body (Figure 9c). The maximum length (ML) of spreading C2C12 cells was measured on day 4 in groups 1–4 for further estimating the performance of the hydrogel inducing and maintaining the cellular spreading (Figure 9c). The results showed that the ML of C2C12 cells in the RGD-clustered cases was significantly higher than that in the homogenous case (Figure 9d), and that differences became more significant when the RGD clustering rate increased.

### 3.5. Cell Spreading, Elongation, and Connection in RGD-Clustering Dextran Hydrogels

**Figure 10:**
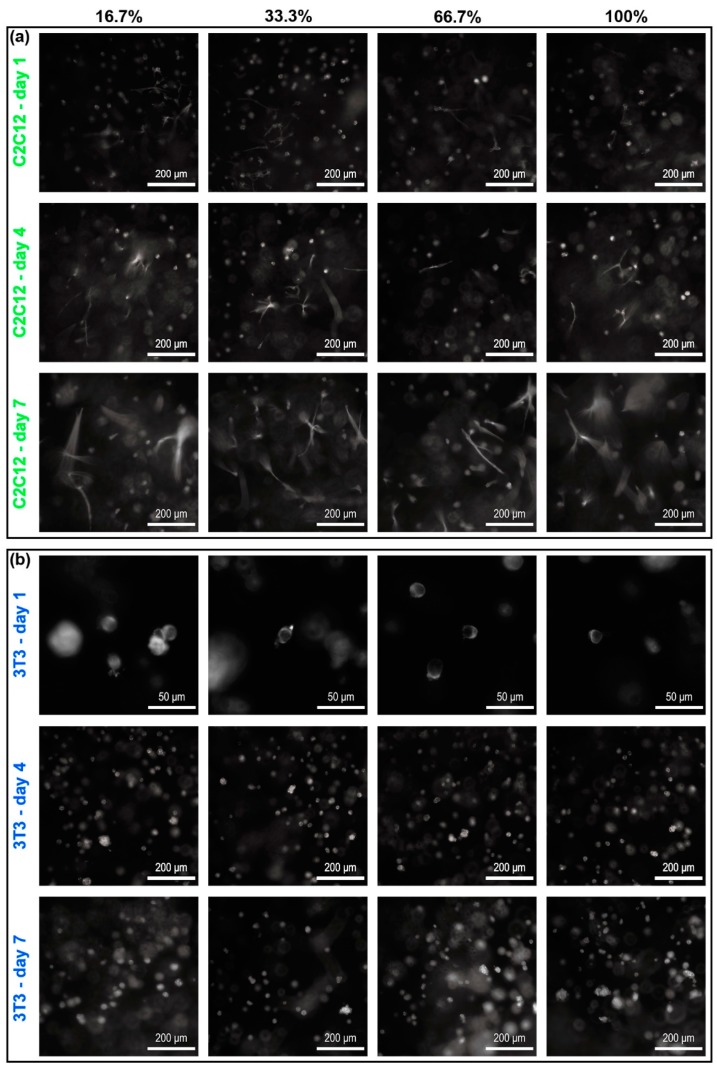
The impact of four different RGD clustering cases on the morphology of F-actin in cells over time. The main parameters of dextran hydrogel were crosslinking strength = 2 mM and RGD = 50 μM. (**a**) Morphology of F-actin in C2C12 cells; (**b**) Morphology of F-actin in 3T3 cells; Cells were observed by an inverted microscope, and the images were taken with a digital sCMOS camera. The proportions of the mal-dextran reacted with RGD for four cases are 16.7%, 33.3%, 66.7%, and 100%, respectively.

**Figure 11:**
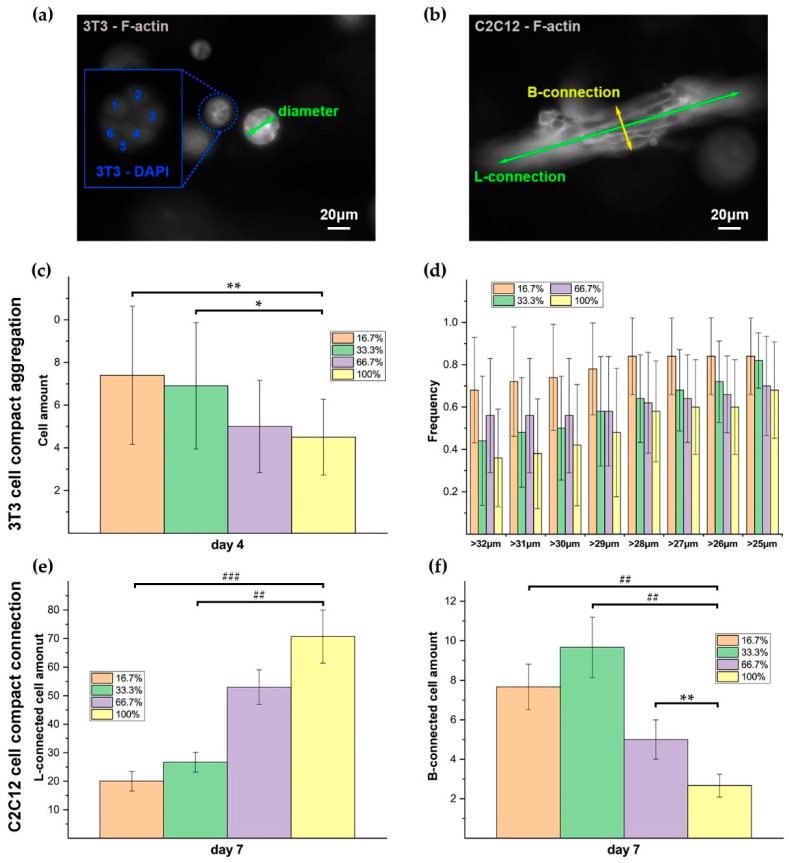
The impact of the composition configuration of 3D dextran hydrogels with clustered and homogenous RGD distribution on cellular collective behaviors. The main parameters of dextran hydrogel were crosslinking strength = 2 mM and RGD = 50 μM. (**a**) The measuring scheme for cell amount of aggregated 3T3 cells and diameter of big 3T3 cell clumps; (**b**) The measuring scheme for cell amounts of L-connected and B-connected C2C12 cells; (**c**) Cell amount of aggregated 3T3 cells on day 4 in groups 1–4; (**d**) Frequency of 3T3 cells growing into big clumps on day 7 in groups 1–4; (**e**) Cell amount of L-connected C2C12 cells on day 7 in groups 1–4; (**f**) Cell amount of B-connected C2C12 cells on day 7 in groups 1– 4. The measurements were conducted by ImageJ software. The data were presented by mean ± SD; * *p* < 0.05, ** *p* < 0.03, ## *p* < 0.003, and ### *p* < 0.001 versus the corresponding 3D homogenous samples.

The elongation and polarization of C2C12 cells and protrusion and aggregation of 3T3 cells were observed (Figure 10a,b). With the extension of culturing time, C2C12 cells came into connections, and the connected multi-cells also showed polarized structures, identical to the single elongated cells (Figure 9c, Figure 10a C2C12-day 7). The cell amounts of C2C12 cells connecting tightly in a path were counted from lengthways (parallel to the polarization) and breadthwise directions (vertical to the polarization) respectively, which were denoted as L-connected and B-connected cells, correspondingly (Figure 11b). The results showed that the amount of L-connected C2C12 increased with the increase of the homogenous level of RGD distribution, and the values were different significantly among the four groups (Figure 11e). However, for the B-connected C2C12 cells, the results of average values were reversed generally, except that there were relatively more B-connected C2C12 cells in the 33.3% RGD clustered dextran hydrogel than that in the 16.7% case (Figure 11f). 3T3 cells tended to aggregated into spherical clumps, which was observed typically in all the four different RGD-clustered 3D dextran hydrogels. The cell amounts of compactly aggregated 3T3 cells were counted on day 4 (Figure 11a), and the results showed that the average values increased with the growing of the clustering level of RGD distribution. Especially, the cell amounts of compactly aggregated 3T3 cells in the 16.7% and 33.3% RGD clustered dextran hydrogels were significantly higher than those in the homogenous case (Figure 11c). Such multicellular clumps grew into bigger scales continuously during cultivation. The frequencies of 3T3 cells growing into big clumps (lower limiting value of diameter ranging from 25 μm to 32 μm) were counted on day 7 (Figure 11a), and the results showed that 3T3 cells grew into bigger clumps in RGD clustering dextran hydrogels (Figure 11d).

## 4. Discussion

In this study, a type of maleimide-dextran hydrogel was used to investigate the evolution of multiplication and self-organization of NIH-3T3 fibroblasts and C2C12 cells in a 3D extracellular matrix compared with the same type of cells in 2D petri dishes. Multipore structures are ubiquitous in gels and work for sufficient flow of the culture medium (Figure 3b). What’s more, it indicated that such hydrogel can help provide a hydrophilic extracellular matrix for soft tissues, which usually require a high water-containing environment. When 3D dextran hydrogel (RGD peptides are homogenous) and a 2D petri dish work as substrate materials, the differences between them could be highlighted by the distinguishing growth phenotypes of cells. We compared the results of NIH–3T3 fibroblasts and C2C12 cells in 2D petri dishes and 3D dextran hydrogel, and found that the 2D substrate and 3D dextran hydrogel stimulated the cells in different manners and the cells responded with different behaviors, which led to the growth of different phenotypes in the corresponding matrices. Additionally, 3T3 and C2C12 cells may interact with the dextran hydrogel in different modes; hence, they behaved differently, and distinguished multicellular structures appeared.

A 3D dextran hydrogel and 2D petri dish provide distinguishing mechanical supports for cells, which could influence the cellular viability differently. The cells in the 2D petri dish always attached to the continuous planar substrate. However, the cells in 3D hydrogel tended to become attached with finite discrete anchor points provided by RGD peptides. Since the total area of substrate for cell attaching often influences cellular viability, it is not surprising that NIH–3T3 fibroblasts and C2C12 cells showed relatively lower survival rates due to the smaller total attaching area in 3D dextran hydrogel. The survival rate and the proliferation, which are typical parameters of cellular viability, also play fundamental roles in the formation of multicellular structures. The cellular survival rate provides solid evidence for evaluation of the biocompatibility of materials. Both 3T3 and C2C12 kept a survival rate of over 75% during the entire test, with an initial cell density of 5 k/μL. This is a positive result in which such hydrogel materials can form a biocompatible substrate and potentially help enhance cellular viability within dense cell transplantation or injection. The substrate materials usually greatly influence the cellular proliferation. We found that the proliferation rates of 3T3 and C2C12 in 3D dextran hydrogel were markedly lower than those of cells in 2D petri dishes. It is consistent with the research results that the 2D substrate with a large enough scale for cell attachment and anchorage can stimulate cellular proliferation effectively [44], which is possibly because a polystyrene culture dish with a large enough bottom surface supported the cells continuously without any other redundant mechanical constrains. Furthermore, in 2D culture, the upper part of cell bodies was completely exposed to the culture medium, which may be beneficial to proliferation. In contrast, the cells in 3D hydrogel were surrounded by a crosslinked dextran polymer. The density of the precursor solution of dextran hydrogel was close to or even lower than the density of cells (Supplementary Figure S1 and Video S1), and thus cells in hydrogel needed another external force to balance the gravity. Adding CD-Link caused hydrogel network formation and cells that were ‘suspended’ in the gel. However, CD-Link may not directly provide forces on cells, because most of the CD-Link did not contact cells directly. Therefore, we inferred that the crosslinked hydrogel with topological networks propped up the cells inside it mainly through the RGD peptide chain on the polymer (Figure 1b). It presents a point-like contact and attachment between the RGD peptide and a cell in 3D, which is much different from the surface attachment manner in 2D culture. This condition in 3D hydrogel increased the cell’s perception of the dangling state, which leads to the decrease of cell proliferation in 3D dextran hydrogel relative to 2D culture [45,46].

Three-dimensional (3D) dextran hydrogel and a 2D petri dish conduct the mechanical stresses on cells distinguishingly, which could influence the cellular spreading and nucleus shape differently. The spreading of cells was often accompanied with the orientation of microfilament bundles [47,48], and usually started at the formation of close contact between cells and the ECM [49]. Previous work has shown that in 2D petri dishes, suspended spherical cells can transform into a fully polarized locomotor state in a few minutes [49]. However, the time of cells spreading in 3D dextran hydrogel was much longer (1 h was the shortest time record in our study). Once settling onto the dish substrate, cells formed attachment gradually, and their microfilaments were almost unconstrainedly and randomly distributed, which allowed the cells to spread freely. When transferred into hydrogel, cells became constrained and their cytoskeletons were under the stress from the surrounding microenvironment. Such stimulus influenced cells’ decisions to spread or to adjust to the suitable cellular shape such as spheres. Once spread and sprouted out, the amount of filopodia of 3T3 in 3D dextran hydrogel was larger than that in the 2D petri dish, which indicates that the hydrogel enhanced the emergence of the filopodia of 3T3 cells; meanwhile, the results of C2C12 were the opposite, which indicates that the hydrogel hindered the emergence of the filopodia of C2C12 cells (Figure 6d). Therefore, such dextran-based biomaterial can potentially provide a platform for distinguishing between 3T3 and C2C12 or even other cells. The dynamic cytoskeletal activities not only led to the variation in cellular morphology, but also caused the change of nucleus shape (Figure 7b,c). The decrease in the circularity of cellular nuclei shown in Figure 7b was possibly due to the conduction of stress from the surrounding cellular cytoskeletons. Cellular cytoskeletons suffered the stress because of cell–matrix interactions such as the cell elongating itself in the elastic dextran hydrogel.

Three-dimensional (3D) dextran hydrogel may help generate the locally inhomogenous distribution of cytokines, which could induce diversified self-organized multicellular structures in such substrate material. Once transferred whether into petri dishes or dextran hydrogels, 3T3 and C2C12 cells were uniformly distributed in the 2D or 3D space (Figure 5, day 1). With the extension of culturing time, cells in 3D dextran hydrogel grew into the structures with an uneven cell distribution (Figure 5, 3D and Figure 7a, 3D). That process was accompanied with cellular proliferation, migration, and apoptosis. However, the distribution of cells in dishes was usually kept nearly constant during the entire cultivation (Figure 5, 2D and Figure 7a, 2D). Although both petri dishes and hydrogel can provide enough adhesion for cellular migration, the cells in them received different biochemical induction. For cells in 2D, at least half of the cytomembrane was exposed to the culture medium directly, and cytokines secreted by cells would quickly dilute into the medium, compared with conditions in hydrogel. Therefore, it was difficult for cells in dishes to decide where to move or which cell to connect with by distinguishing the cytokines coming from almost all 2D directions. That may cause the appearance that the patterns of cells in 2D dishes seemed to be uniform. Cells did move, but their direction could be random. Therefore, the distribution of cells in dishes can be kept nearly constant. For cells in 3D dextran hydrogel, because of the retarding effect of the hydrogels on molecular diffusion [36,50], the cytokines secreted by cells would not completely and immediately dilute into entire extra-environment, but spread into the surrounding 3D space and get weaker gradually. Therefore, cells in hydrogel received cytokines with different strengths from different directions. According to these recognizable cytokines, cells can decide where to spread and which cells to connect with. That may cause the result that cells in 3D hydrogel grew into structures with an uneven cell distribution.

The distinguishing growth performance of the two types of cells gave us quite valued information on the characteristics of interaction between cells and 3D dextran hydrogel compared with that on 2D substrates. Inside the 3D hydrogel, RGD peptides function as the connection between scaffold polymers and integrins on cells. The spatial distribution of RGD peptides in the scaffolds can usually influence cell adhesion dynamics and motility. Here, the specific impact laws of the clustered RGD distribution on the collective cell behaviors in 3D hydrogels, especially for dextran hydrogel, were intensively characterized and analyzed.

The clustering level of RGD peptides on dextran polymer alters the availability of adhesive sites and the adhesion strength on each site, which could finally determine the adhesion efficacy of cells on the polymers. Our data indicated that the 3D hydrogels with different RGD clustering levels gave different influences on the behaviors of both 3T3 and C2C12 cells in each growth stage (day 1, day 4, and day 7). The differences in the distribution of RGD peptides on dextran polymer are responsible for the different adhesion efficacy of the polymers. In the case of 50 μM RGD, the more homogenously the RGD distributes, the more chances for cells to access the attaching sites. Further, in the initial stage just after implantation, the cells may prefer to probe the adjacent RGD anchor for temporary attachment. That may be part of the reason for the 3T3 and C2C12 showing higher initial adhesion rates in homogenous hydrogels on day 1 (Figure 9a,b). After day 1, with the viable cells recovering, their increasing hunger for spreading and migration, especially for C2C12, cannot be satisfied by the sporadic adjacent RGD anchors in the homogenous case anymore. Meanwhile, too many initial cell–matrix conjunctions may reversely become the barriers for the further cellular contraction and elongation during migration in the next stage. The reasons for C2C12 cells that are elongated longer in the highly RGD-clustered hydrogel than that in the homogenous case on day 4 could be explained below. The cell–matrix adhesion strength and the actin stress fiber formation usually depend on the characteristics of RGD ligand presentation, and previous efforts showed that cells on the 2D substrate with particularly clustered RGD tended to exhibit greater elongation and more active migration, which was probably due to the changes of adhesion strength and the actin stress fiber formation [42,51,52]. Moreover, it is possible that cells could feel the adhesion scale, and the clustered RGD peptides could provide more stable conjunctions for cells to stress their fibers on the cell–matrix interfaces [53,54]. That could be the explanation for why C2C12 cells elongated longer in the highly RGD-clustered hydrogel than that in the homogenous case (Figure 9d). In addition, for the cell, integrin clustering usually plays the crucial role in the regulation of the rearrangement of its cytoskeletal structures, which will finally decide the activation and efficacy of its adhesion, migration, and motility [55,56,57]. RGD clustering gives rise to integrin aggregating on the cell–matrix interfaces, and regulates the mechanical forces between the substrate and cells during cell adhesion [51,58]. When designing the substrate materials, it is important to control the spatial distribution and the concentration of the RGD peptides, which will alter the local mechanical properties of the substrate [39,40,41,42]. Further, cells could feel and respond to such local mechanical properties, which will lead to the different cellular behaviors [59,60,61].

The clustered RGD peptides decrease the amount of remaining sites on dextran polymer for crosslinking, which could influence the collective cellular behaviors in the 3D dextran hydrogel, including cell connection and aggregation. When the RGD peptides were clustered, the local stiffness of the dextran hydrogel may alter consequently (we denote it as “stiffness clustering”). That is because for dextran molecules with and without clustered RGD peptides, there are different amounts of maleimide groups left on these two parts of polymers for crosslinking with CD-Link. Therefore, the dextran with clustered RGD peptides has a relatively weaker crosslinking strength than the dextran without RGD peptides, which may cause the decrease of local mechanical strength of the hydrogel, which provided a relative softer substrate for the adhered cells on it [62]. Additionally, previous efforts gave evidence that cells tended to extend their protrusion following the substrate long fibrils along their length, and such an effect would become more obvious when the fibrils were parallel to the direction of the protrusions [61]. When more and more cells adhered on an RGD clustered polymer, the polymer could not offer an adequate balance force for the cellular contraction force, due to the weak mechanical support with less stabilized crosslinking in the local niche with more RGD binding. One of the possible choices of cells is to laterally (perpendicularly to the previous elongation direction) search other anchors for maintaining adhesion and spread. Therefore, C2C12 cells showed more B-connections and less L-connections in higher RGD-clustered dextran hydrogel (Figure 11e,f). Additionally, such locally weak crosslinking may also enhance the aggregation of 3T3 cells. Due to the lowly crosslinking in a local dextran substrate, the hydrogel could provide a looser but more stable 3D substrate for 3T3 cells to aggregate into a bigger scale more easily. Such a “stiffness-clustering” substrate may potentially function as the regulator of the size of 3T3 cell aggregation (Figure 11c,d).

In the homogenous case, because of the homogenous distribution of the RGD peptides, all the dextran polymers were crosslinked evenly. Therefore, the dextran hydrogel with homogenous RGD could provide relatively more mechanical stabilization for collective cell connection. That could possibly explain why C2C12 cells showed more L-connections and less B-connections in RGD-homogenous dextran hydrogels (Figure 11e,f). Additionally, such locally mechanical stable crosslinking may hinder the aggregation of 3T3 cells consequently. Due to the even crosslinking in the local dextran substrate, the hydrogel could provide a stable but too tight 3D substrate for 3T3 cells, which may restrict them from aggregating into a bigger scale.

The 3D RGD-clustered dextran hydrogel usually showed above superiority remarkably when the averaged concentration of RGD was relatively lower (50 μM in this study). When the averaged concentration of RGD peptides in hydrogel increased greatly, the effects caused by clustered RGD would decline obviously. Too many anchors may hinder the active performance of cells in the substrate. We have tested the cases of 300-μM RGD peptides, and the results showed no superiority of C2C12 elongating in the RGD clustering cases after day 4 (Supplementary Figures S2c and S3c). Therefore, the advantages of RGD clustering hydrogel could be amplified by appropriately reducing the RGD concentration according to our results. The dextran hydrogel with clustered RGD composition requires only a much smaller amount of RGD peptide for cellular spread, elongation, and aggregation. Further, the clustering of RGD may induce a more focused rearrangement of skeletal fibers by clustering integrins, and the substrate with clustered RGD could possibly provide more orientated footholds for cell adhesion and retraction. That can potentially become a competitive and economical method for its efficacy of stimulating the spread, elongation, aggregation, and migration of cells.

## 5. Conclusions

The 3D mal-dextran hydrogel with homogenous and clustered RGD compositions provided an extracellular environment with a different external stimulus for cells, and offered flexible and quantitative regulation for the collective cellular behaviors. In this mal-dextran hydrogel material, 3T3 and C2C12 kept stable viability and continuous proliferation, and cells exhibited more tendencies to self-organize into 3D interleaving multicellular structures. Based on this hydrogel material, the behaviors of NIH–3T3 fibroblasts and C2C12 cells can be distinguished by their different growth performance, including spreading, sprouting, agglomeration, and connectivity. The specific cellular behaviors in a 3D dextran hydrogel versus a 2D extracellular matrix might be attributed to the possible stimulus on cells such as external mechanical stress, cytokines with different spreading rates, or the perception of the dangling state. More importantly, our results demonstrate that RGD-clustered mal-dextran hydrogel has the advantage of enhancing C2C12 cell elongation and the breadthwise-aggregated connection, and promoting the 3T3 cell aggregating degree compared to the sample with homogenous RGD. Further, the advantages of RGD clustering hydrogel could be amplified by appropriately reducing RGD concentration, while the RGD-homogenous dextran hydrogels were better at inducing initial cell adhesion. The clustering rate of RGD peptides in this hydrogel could function as the regulator of the cellular growth behaviors such as cell adhesion effect. The reason could probably be attributed to the influence of the RGD ligand distribution on the cell–matrix adhesion strength and the actin stress fiber formation, which then results in the more stable conjunctions for and greater elongation of cells when the clustered RGD peptides are available. That could be the explanation for why C2C12 cells elongated longer in the highly RGD-clustered hydrogel than that in the homogenous case.

## Figures and Tables

**Table 1:** Four different RGD distributions were performed for 3D dextran hydrogel. The levels of RGD clustering decreased from Group 1 to Group 4.

Parameters	Group 1	Group 2	Group 3	Group 4
RGD concentration per gel (μM)	50	50	50	50
Total amount of RGD per gel (nmol)	1.5	1.5	1.5	1.5
% Mal-dextran reacted with RGD	16.7	33.3	66.6	100
RGD clustering (mmol RGD/mmol maleimide group in the first part)	0.1	0.05	0.025	0.017

## Supplementary Materials

The following are available online at https://www.mdpi.com/1996-1944/12/20/3391/s1, Figure S1: Settling process of NIH–3T3 fibroblasts and C2C12 cells in precursor solution of dextran hydrogel, Figure S2: The impact of the composition configuration of 3D dextran hydrogels with clustered and homogenous RGD distribution on C2C12 cellular collective behaviors, Figure S3: The impact of the composition configuration of 3D dextran hydrogels with clustered and homogenous RGD distribution on 3T3 cellular collective behaviors, Figure S4: The proliferation of 3T3 and C2C12 cells in 2D and 3D with initial cell amount of 150,000, Video S1: Slight shake of the few cells rested in the 3D regions above the bottom of the microwell.
