# Supplementary material for: Characterization and Analysis of Collective Cellular Behaviors in 3D Dextran Hydrogels with Homogenous and Clustered RGD Compositions
Source: Materials (Basel). 2019 Oct 17;12(20):3391. doi: 10.3390/ma12203391 (PMC6829236; doi:10.3390/ma12203391)
Supplement: Supplementary file 1 [file materials-12-03391-s001.zip › materials-585880-SI/materials-585880-supplementary.docx]

Supplemental Material

Characterization and Analysis of Collective Cellular Behaviors in 3D Dextran Hydrogels with Homogenous and Clustered RGD Compositions

Zheng Wang ^1,†^, Xiaolu Zhu ^1,2,3,^*^,†^ and Ruiyuan Zhang ^1^

^1^ College of Mechanical & Electrical Engineering, Hohai University, Changzhou 213022, Jiangsu, China; wangzhengd0@163.com (Z.W.); ZRYBrian@163.com (R.Z.);

^2^ Jiangsu Key Laboratory of Special Robot Technology, Hohai University, Changzhou 213022, Jiangsu, China;

^3^ Changzhou Key Laboratory of Digital Manufacture Technology, Hohai University, Changzhou 213022, Jiangsu, China;

***** Correspondence: zhuxiaolu@hhu.edu.cn; Tel.: +86-1586-186-3691

†: These Authors contributed equally to this work.

Received: 16 August 2019; Accepted: 14 October 2019; Published: date

1. The density of the precursor solution

1.1. Methods

In the discussion part of article, we have mentioned that the density of the precursor solution of dextran hydrogel was close to or even lower than the density of NIH–3T3 fibroblasts or C2C12 cells. The validation experiment provided the evidence for that, and the detailed method and results are stated as follows.

Prepare the cell-containing precursor solution as the steps shown in Materials and Methods part of article. Transfer the precursor solution into a 96-well plate and mix it up and down. Then, image the bottom surface of the microwell at an interval of 1 min. By tapping on the table, we conduct the slight vibration on the microwell to detect the distribution of C2C12 cells within the solution during the sedimentation.

1.2. Results and Discussion

During the sedimentation of C2C12 cells, the slight shake of the few cells rested in the 3D regions above the bottom of the microwell indicated the elastic characteristics of the dextran hydrogel (see in an additional video). Image sequences revealed that both NIH–3T3 fibroblasts and C2C12 cells sank to the bottom of the microwell in minutes (Figure S1). It indicated that the density of the precursor solution of dextran hydrogel was close to or even lower than the density of NIH–3T3 fibroblasts or C2C12 cells.


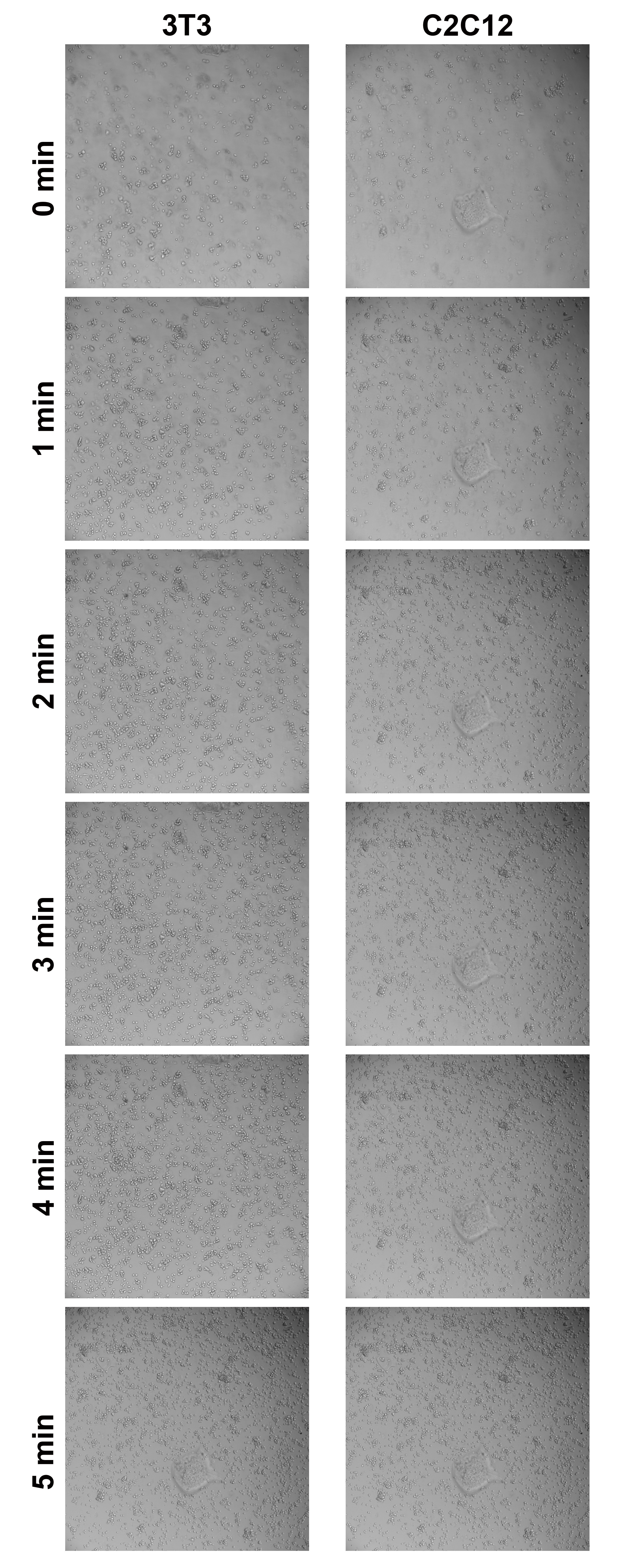


**Figure S1.** Settling process of NIH–3T3 fibroblasts and C2C12 cells in a precursor solution of dextran hydrogel. Images were taken by the inverted biological optical microscope.

2. 300-μM RGD Cases for the RGD-Clustering Dextran Hydrogel Experiments

2.1. Methods

When preparing the precursor solution, maleimide-dextran was divided into two parts for a mixture (Figure 2 in the article). First, DI water, 10 × CB (pH 5.5), the first part of maleimide-dextran, Arg-Gly-Asp (RGD) peptides, and thioglycerol were added into a reaction tube in proportion, mixed thoroughly, and incubated for 5–10 min in room temperature. After the reaction completed, the second part of maleimide-dextran was added into the reaction tube, mixed thoroughly, and incubated for 5–10 min in room temperature. CD-Link was placed onto the bottom of the wells of a 96-well plate. The cell suspension was added into the precursor solution and mixed evenly. The final cell density in the gel was set at 2.5 k/µL. Then, the cell-containing precursor solution was transferred into the wells containing CD-Link and mixed two times quickly and pliably. The hydrogel was completely formed in 3–5 min at room temperature. The sample was covered with fresh culture medium and incubated in the cultivator. The medium was renewed after 2 h of cultivation. Medium was changed every 2 days during cultivation. The volume of each hydrogel sample for cell culture was always maintained at 30 μL, and the reagents were kept on ice. Three different cases were tested, and the related parameter values were listed in Table S1.

For statistics, the sample numbers were all 10, and were used for measuring the maximum length of spreading C2C12 cells (Figure S2b), the frequency of adhered C2C12 cells (Figure S2c), the diameters of aggregated 3T3 cells (Figure S3b), and the frequency of adhered 3T3 cells (Figure S3c).

**Table S1.** Three different RGD distributions were tested for 3D dextran hydrogel. The levels of RGD clustering decreased from Group 1 to Group 3.

| **Parameters** | **Group 1** | **Group 2** | **Group 3** |
| --- | --- | --- | --- |
| RGD concentration per gel (μM) | 300 | 300 | 300 |
| Total amount of RGD per gel (nmol) | 9 | 9 | 9 |
| % Mal-dextran reacted with RGD | 33.3 | 66.6 | 100 |
| RGD clustering  (mmol RGD / mmol maleimide group in the first part) | 0.3 | 0.15 | 0.1 |

2.2. Results

The phalloidin staining results showed that both NIH–3T3 fibroblasts and C2C12 cells could normally spread and sprout in three different RGD clustering cases. Several typical performances of cells were similar with those in the article; the elongation and polarization of C2C12 cells and the slender filopodia sprouting of 3T3 cells were observed (Figure S2a and Figure S3a).


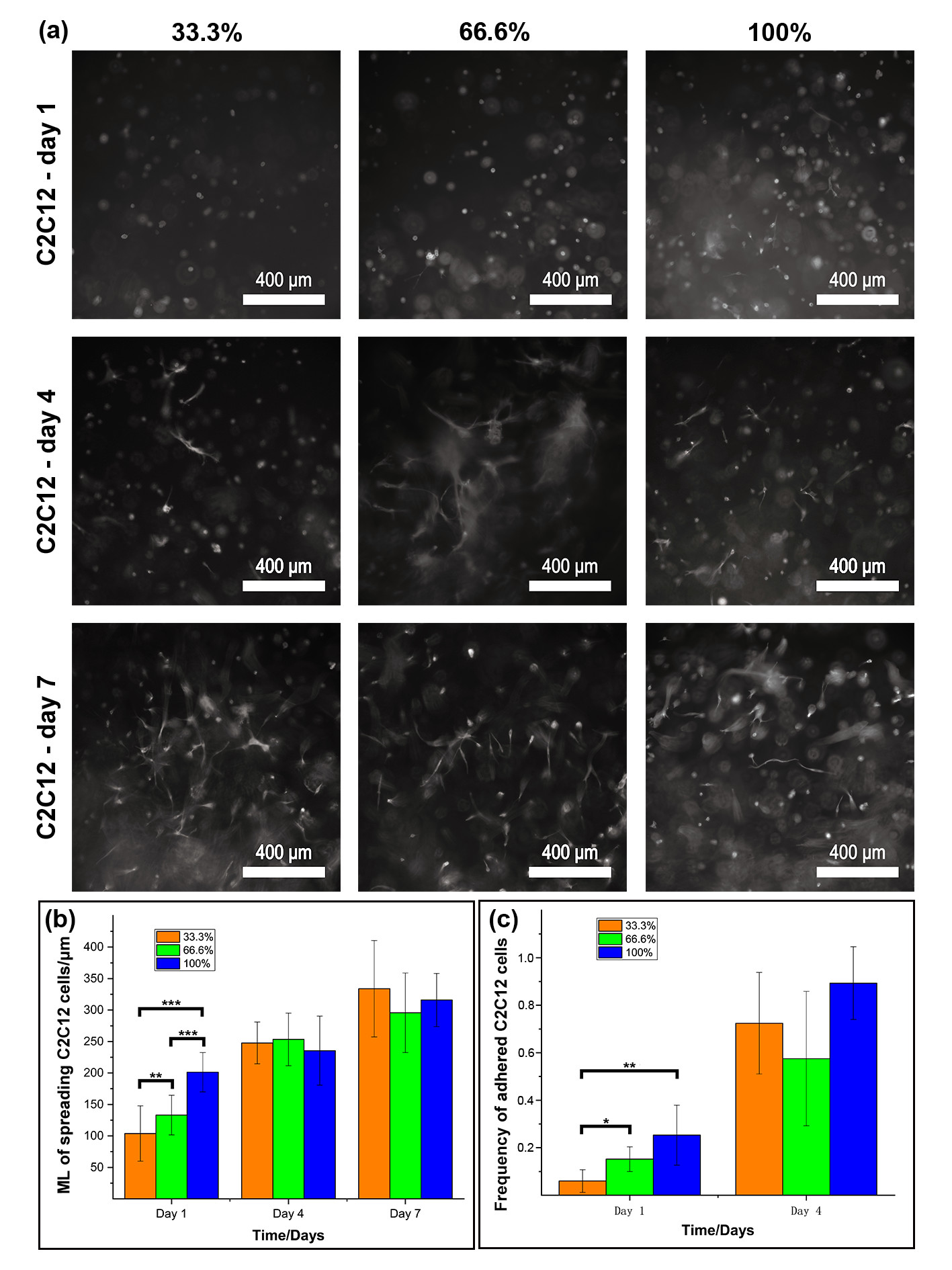


**Figure S2.** The impact of the composition configuration of 3D dextran hydrogels with clustered and homogenous RGD distribution on C2C12 cellular collective behaviors. The main parameters of dextran hydrogel were crosslinking strength = 2 mM and RGD = 300 μM. (**a**) F-actin of C2C12 cells were stained on days 1–7 for four different RGD clustering cases in hydrogels; Cells were observed by an inverted microscope, and the images were taken with a digital sCMOS camera; (**b**) The maximum length (ML) of spreading C2C12 cells was measured on days 1–7 for three different RGD clustering cases. The measurement was conducted by ImageJ software; (**c**) The frequency of adhered C2C12 cells were estimated on day 1 and day 4 for three different RGD clustering cases. The data were presented by mean ± SD; **p* < 0.05, ***p* < 0.03 and ****p* < 0.01 versus the corresponding 3D homogenous samples.


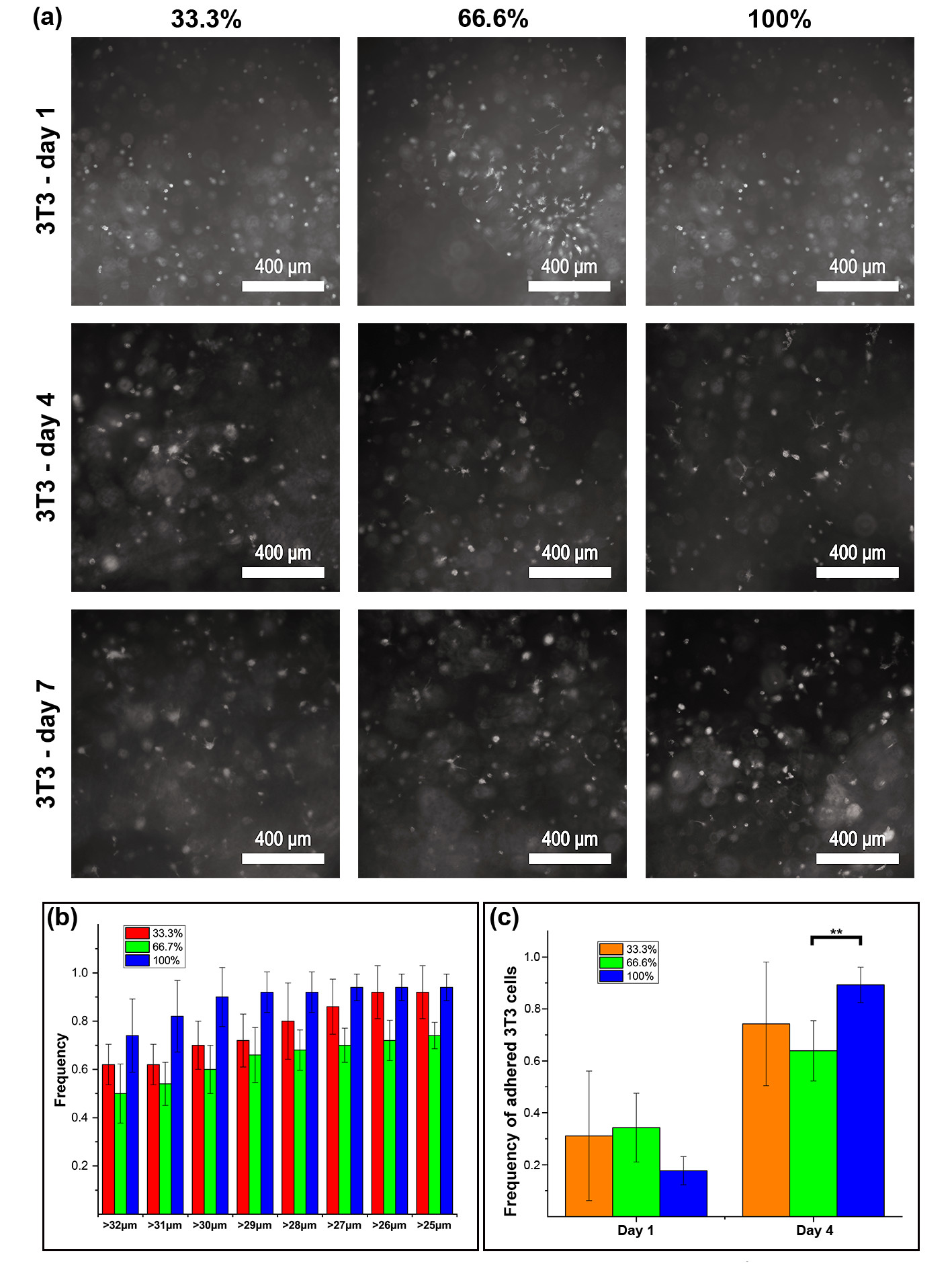


**Figure S3.** The impact of the composition configuration of 3D dextran hydrogels with clustered and homogenous RGD distribution on 3T3 cellular collective behaviors. The main parameters of dextran hydrogel were crosslinking strength = 2 mM and RGD = 300 μM. (**a**) F-actin of 3T3 cells were stained on days 1–7 for four different RGD clustering cases in hydrogels; Cells were observed by an inverted microscope, and the images were taken with a digital sCMOS camera; (**b**) The diameters of aggregated 3T3 cells was measured on days 1–7 for three different RGD clustering cases. The measurement was conducted by ImageJ software; (**c**) The frequency of adhered 3T3 cells were estimated on day 1 and day 4 for three different RGD clustering cases. The data were presented by mean ± SD; ***p* < 0.03 versus the corresponding 3D homogenous samples.

The maximum length of spreading cells was measured in three different RGD clustering cases. On day 1, the length of C2C12 cells was 103.77 ± 44.02 μm in group 1, 133.20 ± 31.54 μm in group 2, and 201.28 ± 31.20 μm in group 3. The results showed that the length value of group 3 was significantly different from that of group 1 and group 2 (*p* < 0.01), and the length value of group 2 was significantly different from that of group 1 (*p* < 0.05). In addition, with the extension of culturing time, all the lengths of the spreading C2C12 cells in three cases increased, and the differences in the length values gradually declined (Figure S2b). The ML of spreading C2C12 cells in group 1 had the largest [increasing](javascript:;) [rate](javascript:;) over the time from day 1 to day 7, compared to the other two groups.

The frequencies of adhered 3T3 and C2C12 cells were estimated in three different RGD clustering cases (Figure S2c and Figure S3c). The results showed that the sprouting rates of cells greatly increased from day 1 to day 4. On day 1, the adhesion rate of the C2C12 cells in group 1 was significantly lower than those in group 2 (*p* < 0.05) and group 3 (*p* < 0.03). With the extension of culturing time, that difference on sprouting rates values gradually weakened. However, on day 4, the adhesin rate of the C2C12 cells in group 3 was relatively higher than that in group 2. In addition, on day 1, the adhesion rate of the 3T3 cells in group 2 was relatively higher than that in group 3. However, on day 4, the sprouting rate of the 3T3 cells in group 3 was significantly higher than that in group 2 (*p* < 0.03).

The diameters of the aggregated 3T3 cells were measured on day 7 in groups 1–3. The results showed that 3T3 cells in the homogenous dextran hydrogels showed higher frequency to aggregate into clumps with diameters over 25–32 μm in general (Figure S3b). The case of 66.7% clustered RGD showed relatively low frequencies in the three groups.

2.3. Discussion

The cellular growth performance can usually be influenced by the cell–matrix interactions. Some research results have shown that the signal molecular clustering can greatly affect cells’ fate in a 2D environment [1,2]. As the anchors for adhesive cells’ attachment, RGD peptides play an important role in the cell spreading and migration [3]. This study tested the 3D dextran hydrogel with different RGD clustering rates for cell culture in vitro. According to the staining results of cellular F-actin, both 3T3 and C2C12 can observably spread and sprout in three different RGD clustering cases. It indicated that such hydrogels can potentially work as biocompatible matrix materials with tunable parameters for inducing cellular behaviors. The cell length values of three groups were significantly different from each other on day 1. It indicated that the different RGD clustering indeed influenced the spreading performance of C2C12 in the initial stage of cultivation. Once the cells have been implanted, hydrogel with homogenous RGD may provide cells with more accessible (or hands-down) surrounding RGD anchors to attach. That may become the reason why the C2C12 length value of group 3 was significantly higher than the others in the homogenous groups on day 1. With the extension of culturing time, cells in different groups gradually formed attachment with each other, and the influence of RGD clustering declined. Therefore, the cell length values tended to be close on the following day 4 and day 7. In addition, the ratio of the average length value on day 7 to that on day 1 in group 1 was 3.22; it was 2.22 in group 2, and 1.57 in group 3. The maximum length of C2C12 in group 1 had the largest [increment](javascript:;) [rate](javascript:;) over the time from day 1 to day 7, compared to other two groups. It may indicate a potential regulation in which the higher RGD clustering rate, the greater C2C12 elongation performed.

3. Cell Proliferation in 2D Six-Well Plates

3.1. Calculation for Suitable Initial Cell Amount

As the control, the distance of each couple of neighboring cells in 2D should be as close as possible to that in 3D, ideally. We assumed that the 3D hydrogel can be approximated as the cube consisting of multi-layer structures, and we set the thickness of each layer to be 30 μm (close to the size of one single 3T3 or C2C12 cell); thus, the 30 μL hydrogel can be divided in to 103 layers. Due to the initial amount of the cells in each hydrogel sample being 150,000, the amount of cells in each layer was 1456. Therefore, on average, each cell in the monolayer took an area of 0.0066 mm^2^, and the average distance of neighboring cells was 81 μm (the square root of 0.0066 mm^2^). Next, we have to calculate the theoretical amount of cells in the 2D petri dish to satisfy that the average distance of neighboring cells in 2D was 81 μm—that is, each cell in the 2D petri dish took an area of 0.0066 mm^2^. Due to the diameter of the 2D wells in the six-well plate we used being approximately 35 mm, the theoretical amount of cells needed should be 146,000 (close to 150,000). Therefore, we set the initial amount of cells in the control group as 150,000.

In 2D culture, for statistics, the well sample (of the six-well plate) numbers used for counting the cell amounts of 3T3 on day 3 and day 6 were 3 and 3; those of C2C12 on day 3 and day 6 were 3 and 3. We conducted the calculation for the mean and standard deviation of the data.

3.2. Results

The proliferations of 3T3 and C2C12 cells in 2D six-well plates and the comparison between 3D and 2D are indicated as below:

Results showed that both 3T3 and C2C12 cells had larger cell amounts in 2D six-well plates than those in 3D homogenous dextran hydrogels on day 3 and day 6, which indicates that 3T3 and C2C12 cells have higher proliferation rates in 2D six-well plates (Figure S4). A partial reason accounting for that had been discussed in section 4 of the original manuscript .


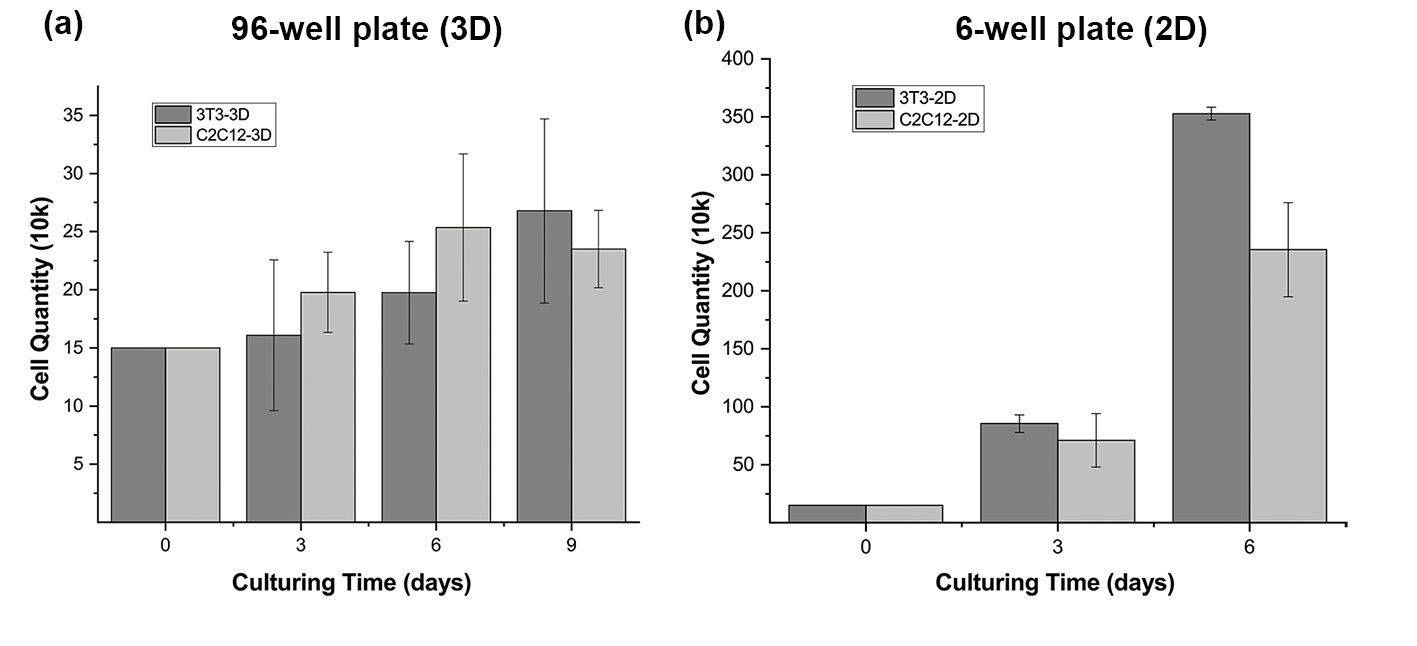


**Figure S4.** The proliferation of 3T3 and C2C12 cells in 3D and 2D with an initial cell amount of 150,000. (**a**) Proliferation of 3T3 and C2C12 cells in 3D homogenous dextran hydrogels in the 96-well plate; (**b**) Proliferation of 3T3 and C2C12 cells on the 2D bottom surface of the six-well plate. The data were presented as mean ± SD.

References

1. Comisar, W.A.; Kazmers, N.H.; Mooney, D.J. Engineering RGD nanopatterned hydrogels to control preosteoblast behavior: a combined computational and experimental approach. *Biomaterials* **2007**, *28*, 4409–4417.

2. Maheshwari, G.; Brown, G.; Lauffenburger, D.A. Cell adhesion and motility depend on nanoscale RGD clustering. *J. Cell Sci.* **2000**, *113*, 1677–1686.

3. Lutolf, M. P.; Lauer-Fields, J. L.; Schmoekel, H. G.; Metters, Andrew T.; Weber, F. E.; Fields, G. B.; Hubbell, J. A. Synthetic matrix metalloproteinase-sensitive hydrogels for the conduction of tissue regeneration: engineering cell-invasion characteristics. *Proceed. Nation. Acad. Sci.* **2003**, *100*, 5413–5418.

© 2019 by the authors. Submitted for possible open access publication under the terms and conditions of the Creative Commons Attribution (CC BY) license (http://creativecommons.org/licenses/by/4.0/).
